# Retinal Progression Biomarkers of Early and Intermediate Age-Related Macular Degeneration

**DOI:** 10.3390/life12010036

**Published:** 2021-12-27

**Authors:** Rita Flores, Ângela Carneiro, Sandra Tenreiro, Miguel C. Seabra

**Affiliations:** 1 Department of Ophthalmology, Centro Hospitalar Universitário de Lisboa Central EPE, 1169-050 Lisbon, Portugal; 2 CEDOC, Chronic Diseases Research Center, NOVA Medical School, 1150-082 Lisbon, Portugal; stenreiro@nms.unl.pt (S.T.); miguel.seabra@nms.unl.pt (M.C.S.); 3 Department of Ophthalmology, Centro Hospitalar Universitário de São João, 4200-319 Porto, Portugal; acarneir@med.up.pt; 4 UCL Institute of Ophthalmology, London EC1V 9EL, UK

**Keywords:** age related macular degeneration (AMD), drusen, color fundus photography, optical coherence tomography, OCT-angiography, progression biomarkers

## Abstract

Early and intermediate AMD patients represent a heterogeneous population with an important but variable risk of progression to more advanced stages of the disease. The five-year progression from early and intermediate AMD to late disease is known to range from 0.4% to 53%. This wide variation explains the particular interest in searching predictive AMD biomarkers. Clinical parameters such as drusen size, presence of pigmentary abnormalities, and fellow eye status were, traditionally, the more important predictive elements. Multimodal retinal assessment (Color Fundus Photography, Optical Coherence Tomography, Optical Coherence Angiography and Fundus Autofluorescence) is providing new and accurate image biomarkers, useful in research and in daily practice. If individual progression risk could be anticipated, then management plans should be adapted accordingly, considering follow-up intervals and therapeutic interventions. Here, we reviewed the most important image progression biomarkers of early and intermediate AMD with relevant interest in clinical practice.

## 1. Introduction

Age-related macular degeneration (AMD) is the leading cause of irreversible severe vision loss in individuals over 55 years old in the developed world [1,2]. The Age Related Eye Disease Study (AREDS) group classifies AMD into three categories based on the type and severity of fundus lesions, including drusen dimensions and pigmentary changes [1,3]: (a) early AMD, defined as the presence of medium drusen (63 to 125 μm) with no pigmentary abnormalities; (b) intermediate AMD, defined as the presence of large drusen (>125 μm) or medium drusen in the presence of pigmentary abnormalities; and (c) advanced AMD, characterized by exudative neovascularization (nAMD) and/or geographic atrophy (GA) [1] involving the center of the macula. A more recently described entity characterized by the presence of new macular vessels in the absence of exudation, designated nonexudative macular neovascularization in naïve AMD patients, should be considered in the intermediate AMD category [4,5,6].

The hallmark of the disease is drusen, which are yellowish-whitish deposits of extracellular debris located between the basal lamina of the retinal pigment epithelium (RPE) and the inner collagenous layer of Bruch’s membrane [7]. Drusen can be classified as hard, soft, cuticular, and pseudo-reticular. Hard drusen are present in 80% of the population over 30 years of age and appear as round, discrete yellow-white spots, measuring less than 63 µm, currently considered as a physiological sign of ageing [7]. Soft drusen are ill-defined, measuring more than 63 µm and containing non-discrete borders. They are also associated with aging, affecting 26% of individuals over the age of 70. Cuticular drusen are yellow deposits located below the basal membrane of RPE, triangular in shape, with a small diameter (50/75 µm) and usually distributed in clusters. Reticular pseudo-drusen or subretinal drusenoid deposits (SDD) are a yellow faint interlacing network located above the RPE, first identified on blue-light fundus photography and sometimes undiagnosed on Color Fundus Photography (CFP) [7,8].

The five year progression from early and intermediate AMD to advanced disease is known to range from 0.4% to 53% depending on disease severity stage [3]. This wide variation in AMD risk progression reflects the population heterogeneity and explains the particular interest in exploring different phenotypes and in searching predictive biomarkers. Since 2017, the Consensus International Group (CAM—Classification of Atrophy Meeting) has been discussing and publishing on this subject, whose main objective is to provide standardized biomarkers for future interventional clinical trials [9,10,11,12,13]. Predictive knowledge is also an important goal in clinical practice, supporting the identification of patients at increased risk of late AMD changes. Evaluation of drusen in color fundus photographs (CFP) was, in the past, the main clinical standard for assessing intermediate AMD patients. There seems to be a positive correlation between the number, area, and extent of drusen observed in CFP and the risk of progression in more than two years [14]. Nevertheless, this method has limited value for predicting individual AMD progression because it is unreliable in the short term [14,15].

More advanced experimental predictive strategies including a combination of the aforementioned drusen classification in CFP combined with genetic, demographic, and environmental factors, such as smoking, light exposure, or diet have been proposed [16]. Some authors have proposed an online risk calculation considering age, family history, smoking habits, fundoscopy, and optional genetic data (Advanced AMD Risk Calculator, http://caseyamdcalc.ohsu.edu/ accessed on 26 December 2021).

While these methods show promising results, they do not explore a variety of quantitative features of drusen and adjacent structures that can be useful predictors of AMD progression [15,17]. The recent widespread use of non-invasive approaches (Optical Coherence Tomography, OCT-angiography, and Fundus Autofluorescence) in early and intermediate AMD retinal imaging has provided important tools in the identification of biomarkers of disease progression. With the aid of such multimodal retinal imaging modalities, complemented with automated algorithms, clinicians and researchers are suggesting a wide range of under-recognized image biomarkers of AMD progression that can be useful and promising in clinical practice.

## 2. Color Fundus Photography (CFP) Findings

**Figure 1:**
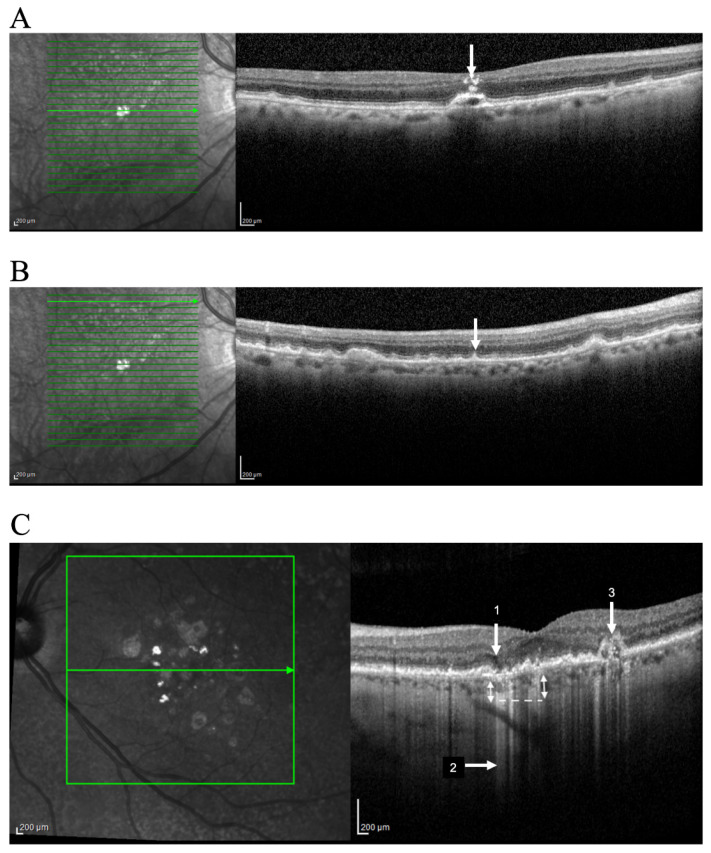
Identification of OCT progression biomarkers of AMD in cross sectional SD-OCT scan. Example of (**A**) Hyperreflective foci (arrow); (**B**) Subretinal drusenoid deposits (arrow); (**C**) iRORA (1); Hyper-transmission defects (2); OCT-reflective drusen substructures (ODS) (3). Representative imagens acquired using OCT SPECTRALIS (Heidelberg Engineering, Heidelberg, Germany).

Drusen characteristics explored in CFP are drusen size, area, and pigmentary changes. Natural history studies have demonstrated that the most important risk factors are advanced AMD in the fellow eye, large, soft, and confluent drusen and AMD-related pigmentary abnormalities [3]. Therefore, some strategies of risk calculation have been proposed based on drusen size, pigmentary abnormalities, and fellow eye status [3,16]. The proposed AREDS simplified scoring system assigns to each eye one risk factor for the presence of 1 or more large (≥125 μm) drusen and one risk factor for the presence of any pigment abnormality. The summation of risk factors in both eyes yields the 5-year risk of developing advanced AMD, in at least one eye, increases in an easily remembered sequence: zero factors, 0.5%; one factor, 3%; two factors, 12%; three factors, 25%; and four factors, 50%. For persons with no large drusen, the presence of medium-size drusen in both eyes is counted as one risk factor (Figure 1A) [3].

Drusen area has been associated with the development of advanced or late stage of AMD [18]. This relationship may result from loss of the overlying photoreceptors and the outer retina layers due to focal disruption or local compression induced by the drusen itself [19,20]. Other studies found that the most important risk factor is the presence of advanced disease in the fellow eye, reporting less significance in drusen size and total drusen area, particularly in the risk of conversion to neovascular AMD [21]. Drusen area can be calculated from manually tracing drusen or by semiautomatic drusen analysis from color fundus images. Those estimates are considerably subjective, depending on aspects related to the observer and related to the patient (opacities, pigmentation, and variability of drusen appearance). Studies comparing drusen area calculation obtained by CFP and by OCT showed only a fair agreement [22,23]. Drusen area measurements tend to be larger with CFP than with OCT. This difference can be attributed to an underestimation of small and flat drusen on OCT measurements.

The AREDS group reported that drusen regression is also a risk factor, as 82% of the eyes that developed significant atrophic changes had preceding drusen regression and hypopigmentary changes [16]. Other researchers further reported that in patients with early to intermediate AMD, drusen regression occurred in 44% of the cases, and preceded the progression to advanced AMD (both GA and nAMD).

Refractile drusen, with calcified nodules, are marked by RPE loss and may represent a possible step before regression and subsequent conversion to advanced stages of the disease [24]. Their glistening appearance results from calcium-containing spherules. In the past, it was supposed that drusen regression caused RPE death, but recent research supports the opposite hypothesis, namely that RPE dies as the drusen collapse [25].

Drusenoid pigment epithelium detachment (PED) results from soft drusen coalescence, reaching diameters >350 μm. Natural history of drusenoid PED presents some overlapping features with the drusen: size and confluence are related to progression. AREDS study reported 19% progressing to GA and 23% to nAMD in 5-year follow-up [16].

## 3. Optical Coherence Tomography (OCT) Findings

The main OCT biomarkers related to progression to advanced AMD include drusen volume, hyperreflective foci (HRF), reticular pseudodrusen or subretinal drusenoid deposits (SDD), incomplete retinal pigment epithelial and outer retinal atrophy (iRORA), hyper-transmission defects, and OCT-reflective drusen substructures (ODS) [9,10,11,12,13,26,27,28].

### 3.1. Drusen Volume

In line with previous studies based on CFP, several investigators using spectral-domain OCT in patients with early and intermediate AMD reported that drusen area, height, and length predicted progression to advanced stages [15]. Eyes with higher baseline drusen volume have an increased risk of progression to either nAMD or GA [18], increase in drusen height is an important risk factor for progression to late atrophic AMD, and drusen length seems to have a positive correlation with the risk of conversion to nAMD [29].

More recent Spectral Domain-OCT (SD-OCT) volume studies provided more comprehensive measures [30,31,32,33,34,35]. Quantitative automatic determinations are available in some devices whose role can be useful in clinical practice. It is possible to automatically distinguish normal eyes from eyes with early and intermediate AMD by identifying total retinal volumes and RPE-drusen complex volumes.

Several studies have used automatic RPE-drusen complex (RPEDC) calculations with SD-OCT devices. Advanced RPE analysis in Cirrus HD-OCT platform provides the clinician with a rapid and reproducible quantitative approach for following disease progression in AMD patients (Advanced RPE analysis with RPEDC abnormal thickening and thinning) [36,37]. The RPEDC abnormal thickening and RPEDC abnormal thinning (RAT) volumes were generated by semiautomated segmentation within a 5 mm diameter macular field: macular OCT drusen volumes and RAT volumes increased significantly in AMD eyes over 2 years [36]. These quantitative SD-OCT biomarkers predict 2-year AMD progression and may serve as useful biomarkers for disease progression. Abnormal thinning of the RPE layer is believed to be an early precursor of atrophic lesions, as well as a marker of progression at the margins of expanding GA [36]. The RAT volume measurement improves specificity in distinguishing eyes with early precursors of GA from healthy control eyes, because control eyes did not demonstrate significant change in this parameter over 2 years [36].

Swept Source (SS) technology, with its longer wave-length and faster acquisition, provides better penetration below the RPE and therefore better visualization of the external layers of the retina and choroid. Improved visualization of external layers with SS-OCT devices, provides better automated segmentation performances, and more concordance in manual and automated measurements of drusen volume. Even more, some authors found no significant differences in drusen volume prior to and after manual correction in SS-OCT compared with a significant difference in SD-OCT devices [38].

### 3.2. Hyper-Reflective Foci

HRF are well defined, highly backscattering lesions within the neurosensory retina; they can be located adjacent to the drusen edge or apex (Figure 1A) or in the inner neurosensory retina. They are described as representing extracellular pigment granules and outer segment debris (outer HRF) or displacement and clumping of degenerated RPE cells or microglia (inner HRF). Curcio et al. hypothesized that anteriorly migrating RPE constitute one population of HRF and posteriorly migrating microglia the other [39].

RPE tends strongly to migrate anteriorly, suggesting either attractive signals from the retina or repellent signals from the drusen or both. One explanation is that cells at the druse apex are at maximum distance from the choriocapillaris and thus migrate into retina to seek oxygen from retinal vessels [39]. Another hypothesis considers that the high mechanical tension on the RPE layer at the drusen apex might serve to eject RPE cells [39].

HRF can appear as single or in clusters. They are relatively stable structures and considered a strong predictor of AMD progression [27]. The number and distribution of HRF across retinal layers are factors with predictive value in development of GA and nAMD, the latter being more associated with inner location and cluster distribution [27]. An extension of the AREDS2 study using SD-OCT demonstrated that patients with HRF on OCT at baseline had 5 times increased risk of progression to GA at 2 years when compared with controls [27].

Correlation with neovascular evolution is not so well demonstrated. Nevertheless, in nAMD, the regression of HRF after anti-VEGF treatment is considered a good functional prognostic sign. It was demonstrated that one month after the first injection, there was a statistically significant regression of HRF (*p* < 0.04) and an even more significant regression at the 3-month follow-up examination (*p* < 0.01). In the same study, there was a statistically significant reduction of HRF in the good and moderate visual acuity subgroups (respectively, *p* < 0.01 and *p* < 0.02). The persistence of HRD correlated significantly with the poor visual acuity group (*p* < 0.02) [40].

### 3.3. Reticular Pseudodrusen

These subretinal collections of granular, interlacing, hyper-reflective material located above RPE are commonly found in the superior macula or close to superotemporal arcades (Figure 1B). Observable by OCT, reticular pseudodrusen or SDD undergo a characteristic lifecycle of growth, invasion into the adjacent ellipsoid zone, and finally regression. Advanced stages in the SDD lifecycle are related to RPE degeneration and photoreceptor loss [41].

Infrared reflectance (IR) and SD-OCT appear to be particularly relevant methods to diagnose SDD. Using multimodal imaging, the prevalence of SDD appears higher than previously reported in studies based on retinal photography only. Regarding imaging techniques, the prevalence of SDD is lowest when diagnosed by CFP (6.7%) and fundus autofluorescence FAF (9.5%) and reaches the higher percentages when diagnosed with IR (18.1%) or SD-OCT (17.4%) [8].

Reticular pseudodrusen are found to be present in approximately 9% to 58% of patients with intermediate AMD, depending on the population studied [42,43]. In other studies, reticular pseudodrusen were present in 4.6% of eyes without AMD, 13.0% with early AMD, 62.6% with intermediate AMD, 34.6% with atrophic AMD, and 8.1% with neovascular AMD [8,42]. Researchers reported that the presence of reticular pseudodrusen is associated with an additional 2 to 6-fold increased risk of progression to nAMD or central GA, with the risk of progression higher for reticular pseudodrusen located outside the macula [42].

### 3.4. Incomplete Retinal Pigment Epithelial and Outer Retinal Atrophy

The term iRORA refers to the localized loss of tissue in the outer layers of retina without definite RPE loss. iRORA represent the new nomenclature obtained by the Consensus International Group (CAM—Classification of Atrophy Meeting) and has, more or less, replaced the so called “Nascent GA” [9,10,11,12,13]. Nascent GA and iRORA have not exactly the same meaning, because nascent GA should be only used in the absence of current or prior macular neovascularization.

iRORA represents a subsidence of the outer plexiform layer and the inner nuclear layer with a hypo-reflective wedge (Figure 1C) [44]. This finding is found in 22% of eyes with intermediate AMD and in 7% of all AMD patients [45]. iRORA is defined on OCT as: (1) a region of signal hypertransmission into the choroid; (2) a corresponding zone of attenuation or disruption of the RPE; and (3) evidence of overlying photoreceptor degeneration. The term iRORA should not be used when there is an RPE tear [11]. iRORA is strongly associated with impending GA as an average of 11 months elapsed between early signs of nascent GA and the development of GA [45].

Recognizing that photoreceptor atrophy can occur prior to RPE atrophy, and that atrophy can undergo different stages from outer retinal layer to RPE involvement, two previous stages were described in the atrophic progression: incomplete outer retinal atrophy (iORA) and complete outer retinal atrophy (cORA). This new classification opens novel reflection data in the atrophic pathologic development with progressive and complementary involvement of RPE and outer retinal layers cells.

In this new nomenclature, the term complete RPE and outer retinal atrophy (cRORA) was proposed as an end point for atrophy that occurred in the presence of drusen and was defined by the following criteria: (1) a region of hypertransmission of at least 250 mm in diameter; (2) a zone of attenuation or disruption of the RPE of at least 250 mm in diameter; and (3) evidence of overlying photoreceptor degeneration, all occurring in the absence of signs of an RPE tear [10].The CAM group believed that the term GA, well known in the AMD nomenclature and with several current CFP definitions, should remain and continue to be used as a term applied to the subset of cRORA occurring in the absence of current or past macular neovascularization [10].

Multimodal retinal imaging allows earlier and precise diagnose of atrophic AMD progression compared with isolated CFP. OCT identification of these different precursor lesions is an important issue in understanding the evolution to atrophic late stages [46].

### 3.5. Hyper-Transmission Defects

A commonly described OCT feature associated with atrophy is the presence of increased transmission of signal below the level of the RPE and into the choroid, resulting from loss of scatter or attenuation from overlying RPE and neurosensory retina.

A variety of terms were proposed including sub-RPE illumination, choroidal hyperreflectivity, narrow columns of hyper-reflectivity, and hyper-transmission defects. CAM participants agreed that the term hyper-transmission defects was the preferred term because it reports to the cause for the observed phenomenon and includes all cases even those with tall PEDs, where the hyper-transmission may not always penetrate to the underlying choroid [9,10,11,12,13].

Another common feature was a persistent hyperreflective line within the atrophy area, but significantly thinner than the adjacent RPE Bruch’s membrane band. This line is supposed to represent the so called “persistent basal laminar deposit” corresponding to a few dissociated RPE cells, as well as RPE granules, processes of Müller cells, and avascular fibrosis [47].

Finally, we can observe narrow columns of hyper-reflectivity under the RPE that suggest deficiencies in the RPE layer (Figure 1C) [48]. Prevalence is still unreported but some authors described sub-RPE hyper-reflective columns in 27% of eyes that progressed to nAMD or GA by at least three months [48]. These hyper-reflective columns are essentially hypertransmission in narrow strips, and their potential different meaning is not well defined.

### 3.6. OCT-Reflective Drusen Substructures

Variation in the OCT structure and properties of drusen has also been proposed to represent an increased risk of AMD progression. Drusen usually appear as a smooth, dome-shaped RPE elevation with homogeneous medium internal reflectivity and without HRF. These characteristics occur in about 47% of all drusen and only in 50% of the so-called soft drusen in CFP [17]. However, varying morphology, reflectivity, and internal homogeneity may be observed in other cases and these findings are important in monitoring intermediate AMD patients (Figure 1C).

A large multicenter study investigated such variations in patients with intermediate AMD enrolled in the AREDS2 SD-OCT study [49]. The authors found drusen substructures in 24% of intermediate AMD patients and described four phenotypic subtypes of variations or ODS: (a) low-reflective core (L type); (b) split-reflective core (S type); (c) high-reflective core (H type); and (d) conical debris (C type).

Presence of ODS at baseline in eyes with intermediate AMD was associated with progression to GA, but not to nAMD [49]. Low reflective cores may consist primarily of lipids, whereas high reflective cores may consist primarily of proteins and hydroxyapatite. Split drusen may simply be a heterogeneously composed drusen, with disease-associated molecules that have not seeded onto spherules, hence the split designation [49]. Conical debris may be calcified deposits caused by a shattering of high reflective cores. Conical debris (C type) is the subtype more closely related to atrophic changes, followed by H type, S type, and finally by L type [49].

In more recent studies, authors analyzed the heterogeneous internal reflectivity material within drusen (HIRD) and concluded it is formed by multilobular nodules composed of hydroxyapatite and that they are quite different from the spherules or refractile drusen, another refractile feature found in the retinas of patients with AMD [50]. Unlike spherules, which are small, refractile on CFP and reflective on OCT, nodules are large, refractile on CFP and nonreflective on OCT. Nodules are not simply aggregations of spherules and the heterogeneous HIRD should be called “calcified nodules” instead of the alternative terms “hyperreflective pyramidal structures” or conical debris [50]. These findings suggest that hydroxyapatite nodules may be indicators of progression to advanced AMD and that multimodal retinal imaging can help us to find outcome measures in AMD progression [50].

### 3.7. Other OCT Morphologic Findings

Ellipsoid zone disruption and focal RPE disruptions have also been associated with progression to advanced AMD and neovascularization [51]. Other features, independently associated with progression to advanced AMD, include presence of drusenoid PED, presence of RPE thickening, focal irregularity of the retina (thickening or thinning), irregularity or disruption of the external limiting membrane, and choroidal vessel abnormalities noted on OCT [10]. Table 1 presents a summary of the most common OCT progression biomarkers in AMD.

## 4. Optical Coherence Tomography-Angiography Findings

OCT angiography (OCT-A) provides a non-invasive method by which clinicians and researchers can diagnose and monitor exudative and nonexudative neovascularization in AMD eyes [4,5,6].

Non exudative neovascularization demonstrated ill-defined hyperfluorescence with no leakage on fluorescein angiography, hypercyanescence in a plaque configuration on indocyanine green angiography, and a shallow, irregular pigment epithelial detachment with moderate hyperreflectivity on OCT [52,53]. The detection of nonexudative neovascularization increased significantly with OCT-A imaging and actually evidence of nonexudative neovascularization occurs in about 6.25% to 27% in the fellow eye of exudative AMD [54].

Naïve patients with nonexudative neovascularization are phenotypic intermediate AMD patients and can be classified as subclinical or quiescent neovascularization (stability beyond six months). They carried an increased risk of conversion to exudative AMD compared with patients who did not have nonexudative neovascularization (21.1% vs. 5.4% at one-year follow-up) [5,52,54].

Early detection of macular neovascularization vessels should result in better early monitoring of patients with intermediate AMD who are at high risk of conversion to late neovascular AMD. Until data from natural history studies are more consistent, eyes with non exudative neovascularization should be followed up more closely than eyes without neovascularization because of the presumed increased risk of exudation and vision loss [52]. The possibility to include OCT-A as an imaging technique to be performed at baseline and at follow-up visits of intermediate AMD should be considered in the near future, as long as some limitations that preclude the use of OCT-A in daily practice are minimized. Anti-VEGF treatment is not recommended in those cases, until the appearance of signs of exudative activity [52]. Some studies even consider the possibility of a potential protective effect on overlying neurosensory retina, probably related with improved oxygenation [6,58]. Investigators are interested in exploring anatomic changes related with near-term exudation. The onset of exudation seems to be correlated with lesions with less vascularity and smaller pigment epithelium detachment volume. Other parameters as macular neovascularization area, choroidal thickness, choroid vascularity index, and choriocapillary flow deficits were also studied, with no proved relationship with new exudation [59].

Nonexudative neovascularization needs differentiation from the so-called inactive neovascularization. Inactive neovascularization is defined by absence of exudative activity (6 months with no fluid on OCT) in a previously treated neovascular lesion. As opposed to quiescent or subclinical neovascularization, those lesions had been active and treated in the past. So, this term does not apply to naïve neovascularization and is documented in OCT-A as a skeletonized net [52,60,61].

## 5. Fundus Autofluorescence (FAF) Findings

The more important FAF findings to highlight in early and intermediate AMD are FAF patterns of drusen, of SDD, and those associated with pigmentary changes. SD-OCT imaging largely overcomes conventional FAF as it adds insight into the condition of the neurosensory layers and associated alterations at the level of the RPE and choroid [62]. In addition, FAF imaging modalities are more influenced by opacities than SD-OCT.

Drusen FAF patterns were related to histologic examination, in the first direct clinicopathologic study correlation. Authors described four FAF stages as follows [62]. Stage 1 is characterized by isoautofluorescent drusen (visible on CFP); stage 2, with mildly uniform hyperautofluorescence corresponding to short photoreceptors suffering and complete coverage by RPE; stage 3, where a ring of hyperautofluorescence is visible around an hypoautofluorescence center corresponding to early RPE atrophy (RPE gaps); stage 4, with a uniform hypoautofluorescence corresponding to absence of RPE (complete RPE atrophy), and which includes a high proportion of refractile drusen (82%) with many calcific nodules, visible on CFP.

Stages 3 and 4 associate with incomplete RPE and iRORA as defined by the CAM group. Loss of RPE, the outer nuclear layer, and the external limiting membrane ELM in stage 4 indicates that atrophy can begin over individual drusen.

Reticular pseudodrusen are identified in FAF imaging as an area of isoautofluorescence on the center of each reticular pseudodrusen surrounded by halos of reduced autofluorescence, responsible for the “target aspect”, around the macula and, especially, along the superior vascular arcades [8].

Pigmentary abnormalities also promote FAF changes whose patterns depend on the presence of hypo or hyperpigmentation and on the status of RPE cells.

FAF patterns are largely studied in atrophic AMD, particularly in the junction zone [63], but the use of this imaging modality in intermediate AMD has not defined a particular FAF pattern exclusively associated with conversion or progression of intermediate AMD [64].

## 6. Conclusions

Although routine ocular imaging in AMD clinical assessment is useful and indispensable, there is still no accurate, single or in combination, biomarker that is able to reliably identify those eyes that will progress from those that will not. Nevertheless, as AMD progression carries a significant negative visual prognosis, and its detection has important clinical implications. Not only do predictive biomarkers provide insights into AMD progression, but they may also prove to be valuable in managing and monitoring patients with this disease.

The ideal risk calculator should integrate a combination of demographic, historical, genetic, functional, and structural risk factors accumulated over time. Theoretically, it should be able to provide a clinically meaningful and accurate absolute risk score. Analogous to the experience with risk prediction in cardiovascular disease, these tools should require greater scrutiny of predictive performance, internal and external validation, and head-to-head comparison; unfortunately, they are still not available for practical use.

Clinicians must take into consideration the level of associated risk and the strength of the evidence of each particular risk factor (Table 2). Although there is no current consensus strategy for identifying patients at greatest risk of progression, we can use the available evidence to better stratify and manage patients with intermediate AMD.

In this prognostic evaluation, we should consider the information obtained by still-useful clinical criteria: presence of late AMD fellow eye, large drusen, and pigmentary abnormalities. Despite its potential high clinical usefulness, the ability to predict progression from intermediate to late AMD requires full patient phenotyping obtained by multimodal imaging including CFP, OCT, OCT-A, and FAF [45].

Important and recent studies have documented significant and interesting correlation between histology and OCT findings in AMD [56,66,67]. The association of OCT with other retinal image techniques increases diagnostic precision even more.

Regarding OCT progression biomarkers, the main OCT features associated with increased risk of progression to late AMD are HRF, SDD, and drusen volume. As for progression to nAMD, only HRF and SDD were clearly associated [68].

Some authors purpose to develop a simple, clinical, and practical OCT-based scoring system for early and intermediate AMD to prognosticate risk progression to late AMD; those algorithms still need validation [68,69].

Machine learning and artificial intelligence are promising tools to image guided prediction of AMD progression. Large data and instant analysis would accelerate and contribute to identifying new biomarkers of disease progression [70,71,72].

Interventions that stop or delay the transition from early and intermediate to late AMD are acutely needed. Randomized clinical trials are being conducted to investigate future treatments, potentially useful in early and intermediate stages of the disease.

Until effective therapies are recommended with probed efficacy, management plans should be tailored to the level of risk patients with a higher probability of progression, considering more regular surveillance, smoking cessation, self-monitoring strategies, and nutritional supplements.

## Figures and Tables

**Table 1:** Progression biomarkers in AMD.

Biomarker	Imaging Findings	Mechanism(s)	Prevalence in AMD%	Expected Progression (OR ^1^)
Drusen volume	Baseline drusen volume	Displacement or deterioration of photoreceptor layer	ND ^2^	1.31 risk of progression to nAMD (for each 0.1 mm^3^ of drusen volume increase) [36]
RPE-Drusen complex (DC) Advanced analysis	RAT ^3^	RPE suffering and drusen regression	ND ^2^	1.32 risk of developing central GA (for each 0.001 mm^3^ increase in RAT volume) [36]
HRF	Punctate hyperreflective lesions	Anterior migration of fully pigmentated RPE cells, inflammatory or microglia cell and calcification	50% in AMD	5 risk of 2-year progression to GA [27]
SDD	Small yellow deposits: reticular, ribbon-like or interdigitated	Dysfunction of cholesterol homeostasis, retinoid processing or choroidal hypoxia [55]	32% to 79% in AMD patients	2.24–3.4 risk of progression to advanced disease [1,42]
iRORA	Subsidence of the OPL ^4^ and INL ^5^ with a hypo-reflective wedge	New onset of atrophy (nascent atrophy)	7% in intermediate AMD [56]	5.2 risk of progression to central GA [45]
Hypertransmission	Columns or strips of hyperreflectivity	Deficiencies within RPE layer	27% in AMD patients [48]	ND
ODS	Internal heterogeneity	Metabolic instability	24% in soft drusen	5.6 risk of progression to new atrophy onset [57]
Non exudative Retinal neovascularization	Neovascular lesion with no fluid	Protective mechanism against ischemia	6.25 to 27% in the fellow eye of exudative AMD [54]	1.21 risk of progression to exudative AMD at 1 year [52]

^1^ Odds ratio; ^2^ not determined; ^3^ RPE Abnormal thinning; ^4^ Outer Plexiform Layer; ^5^ Inner nuclear Layer.

**Table 2:** Prognostic biomarkers for progression in intermediate AMD.

		Strength of Evidence	
		High	Low
**Strength of Risk**	**High**	**HRF** OR ^1^ = 4.72 to central GA, 2 years 95% CI ^2^: 2.43–9.80, *p* < 0.001 [39] **SDD** OR ^1^ = 2.64 to late AMD, 2 years 95% CI ^2^: 1.07–6.49, *p* = 0.034 [42]	**OCT drusen substructures** OR ^1^ = 5.614 for new atrophy onset, 6 months 95% CI ^2^: 1.277–24.673, *p* < 0.001 [57] **iRORA + HRF** OR ^1^ = 10 to cRORA, 2 years 95% CI: 2.36–42.20, *p* = 0.002 [45,65] **iRORA + SDD** OR ^1^ = 1.9 to cRORA, 2 years 95% CI: 0.60–6.2, *p* = 0.263 data [45,65]
	**Low**	**Drusen volume** OR ^1^ 1.31 to late AMD, 2 years 95% CI ^2^: 1.06–1.63; *p* = 0.013 [13] **RPE Drusen Complex** OR ^1^ = 1.32 to central GA, 2 years 95% CI ^2^: 1.14–1.53; *p* < 0.001 [27]	**Hypertransmission** OR ^1^ ND ^3^ [48] **Ellipsoid zone disruption** OR ^1^ ND ^3^ [51]

^1^ Odds ratio; ^2^ confidence interval; ^3^ not determined.

## Data Availability

Not applicable.
